# Feasibility of novel adult tuberculosis vaccination in South Africa: a cost-effectiveness and budget impact analysis

**DOI:** 10.1038/s41541-022-00554-1

**Published:** 2022-11-07

**Authors:** Sahan Jayawardana, Chathika K. Weerasuriya, Puck T. Pelzer, Janet Seeley, Rebecca C. Harris, Michele Tameris, Dereck Tait, Richard G. White, Miqdad Asaria

**Affiliations:** 1 Department of Health Policy, London School of Economics (LSE), London, UK; 2 TB Modelling Group, TB Centre and Centre for the Mathematical Modelling of Infectious Diseases, Department of Infectious Disease Epidemiology, Faculty of Epidemiology & Population Health, London School of Hygiene & Tropical Medicine, London, UK; 3 KNCV Tuberculosis foundation, Hague, Netherlands; 4 Department of Global Health and Development, London School of Hygiene and Tropical Medicine, London, UK; 5 South African Tuberculosis Vaccine Initiative (SATVI), Department of Pathology, Institute of Infectious Disease and Molecular Medicine, University of Cape Town (UCT), Cape Town, South Africa; 6 Independent consultant, Cape Town, South Africa; 7 Present Address: Sanofi Pasteur, Singapore, Singapore

**Keywords:** Tuberculosis, Vaccines

## Abstract

Early trials of novel vaccines against tuberculosis (TB) in adults have suggested substantial protection against TB. However, little is known about the feasibility and affordability of rolling out such vaccines in practice. We conducted expert interviews to identify plausible vaccination implementation strategies for the novel M72/AS01_E_ vaccine candidate. The strategies were defined in terms of target population, coverage, vaccination schedule and delivery mode. We modelled these strategies to estimate long-term resource requirements and health benefits arising from vaccination over 2025–2050. We presented these to experts who excluded strategies that were deemed infeasible, and estimated cost-effectiveness and budget impact for each remaining strategy. The four strategies modelled combined target populations: either everyone aged 18–50, or all adults living with HIV, with delivery strategies: either a mass campaign followed by routine vaccination of 18-year olds, or two mass campaigns 10 years apart. Delivering two mass campaigns to all 18–50-year olds was found to be the most cost-effective strategy conferring the greatest net health benefit of 1.2 million DALYs averted having a probability of being cost-effective of 65–70%. This strategy required 38 million vaccine courses to be delivered at a cost of USD 507 million, reducing TB-related costs by USD 184 million while increasing ART costs by USD 79 million. A suitably designed adult TB vaccination programme built around novel TB vaccines is likely to be cost-effective and affordable given the resource and budget constraints in South Africa.

## Introduction

Tuberculosis (TB) has killed more people globally than any other single infectious disease over the last decade. A vaccine—bacille Calmette-Guérin (BCG)—has existed since the 1920’s, delivered routinely to neonates worldwide, preventing extrapulmonary TB, disseminated TB, and severe TB in children^1–3^. However, most of the global TB burden is in adults^4^, where the efficacy of neonatal BCG is lower^2^. Despite routine use of BCG and drug therapy, the decline in TB burden remains slow and inadequate to achieve global TB control goals^4^, and the COVID-19 pandemic may have slowed progress against TB^5^. As such, new vaccines to prevent adolescent and adult tuberculosis are urgently needed. In 2018, a phase IIb trial of the novel vaccine candidate M72/AS01_E_ showed 50% [95% confidence interval (CI): 2–74%] efficacy in preventing pulmonary tuberculosis disease in *Mycobacterium tuberculosis* (*M. tb*)-infected 18–50 year olds^6^. M72/AS01_E_ is now progressing to a phase III trial, and ultimately may serve as an effective adjunct to neonatal BCG. BCG revaccination in adolescence is also being explored^7^ as a potential avenue, with other candidates at varying levels of progress in the development^8^ pipeline.

Previous modelling studies have estimated the epidemiological impact of hypothetical or pipeline vaccines on tuberculosis infection and/or disease^9–11^, including on drug-resistant tuberculosis, and when delivered via a combination of routine immunisation of 9-year olds and recurring mass campaigns to adolescents/adults^12,13^. Routine vaccination of adolescents (10-, 15-, or 18-year olds) has been predicted to be cost-effective in South Africa and India^14^. However, important gaps in the literature, particularly with regards to vaccination of adults, remain.

First, most analyses construct vaccine implementation scenarios by extrapolating experience from other vaccine programmes. These assumptions may not reflect what is considered feasible or preferable by country decision makers, nor reflect in-country priorities. Second, previous studies have not assessed the economic impact of vaccinating adults with an M72/AS01_E_-like vaccine against TB infection or disease. Third, few studies have actively explored vaccine targeting to key population subgroups, including to people living with HIV (PLHIV). Globally, HIV is the most important risk factor for tuberculosis^4^: the immunodeficiency associated with HIV increases the risk of TB disease following infection and immunocompromised patients suffer substantially worse TB outcomes. WHO estimates that South Africa suffered 328,000 (uncertainty range[UR] 230,000–444,0000) incident TB cases in 2021^15^, of which 234,000 (UR 164,000–316,000) were in PLHIV. Given this, estimating the relative cost-efficiency of targeting this group remains a vital unanswered question.

In this study, we conducted interviews with key experts to explore potential implementation strategies for the M72/AS01_E_ vaccine in South Africa and elicit constraints and preferences. We then assessed the population-level health impact, cost-effectiveness and budget impact of the M72/AS01_E_ vaccination implementation strategies elicited from expert interviews, using a combined epidemiological and economic model. This study is a necessary and timely guide to investment in phase III and IV trials, and trial design and implementation decisions.

## Results

### Selection of vaccination strategies

From the first round of interviews, we identified six potential TB vaccination implementation strategies for South Africa (Supplementary Table 1).

Following the second round of interviews, after taking into consideration the constraints and preferences expressed by the experts, two potential target populations and two ways of delivering the vaccination programme were identified giving us four possible vaccination implementation strategies that we modelled (Table 1). For the Mass&RoutineAdultsHIV+ and 2xMassAdultsHIV+ strategies, we assumed vaccination of all PLHIV (on and off ART), up to the specified routine and mass vaccination coverage. We assumed the vaccination status of PLHIV did not interact with ART compliance.

**Table 1:** Vaccination strategies.

		Target population	
		All 18–50 year olds^a^	All PLHIV adults^b^
Programme delivery	One mass campaign in 2025 followed by routine annual vaccination of 18-year olds	Mass&Routine18–50	Mass&Routine AdultsHIV+
	Two mass campaigns in 2025 and 2035	2xMass18–50	2xMassAdultsHIV+

For the scenario targeting all 18–50-year olds, we assumed 60% coverage for the mass campaigns and 40% coverage for annual routine vaccination of 18-year olds. For the scenario targeting PLHIV adults, 60% coverage was assumed for the mass campaign(s) and 70% coverage for annual routine vaccination of 18-year-old PLHIV.

^a^Representing targeting people aged 18–50 and delivering vaccines via colleges, workplaces and local clinics.

^b^Representing targeting PLHIV and delivering vaccines via clinics providing antiretroviral therapy (ART).

### Epidemiologic impact

**Fig. 1:**
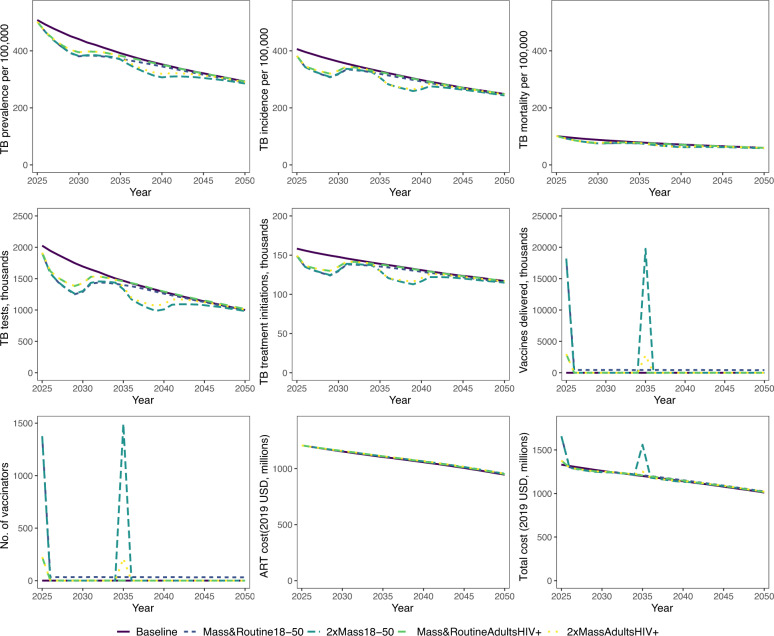
TB health outcomes, resource use and costs for the baseline and 4 vaccination strategies. Mass&Routine18–50—mass campaign for people aged 18–50 and routine vaccination for 18-year olds; 2xMass18–50—two mass campaigns for people aged 18–50; Mass&RoutineAdultsHIV+—mass campaign for all adults living with HIV and routine vaccination of 18-year olds living with HIV; 2xMassAdultsHIV+—two mass campaigns for all adults living with HIV. Lines represent median annual values. Number of total TB notifications from 2025 to 2050 estimated by transmission model. Number of people tested was calculated using an estimated Test-to-Diagnosis (TDR) ratio for South Africa in 2025. A TDR value of 12.8 was used based on Xpert test (primary diagnostic tool used in South Africa) results data from the South Africa National Health Laboratory Services. This value was adjusted for subsequent years by the prevalence of active tuberculosis. A vaccine course was assumed to comprise two vaccine doses. Number of vaccinators estimated assuming that the vaccinations will be delivered throughout the year and 5 minutes of vaccinator time per dose.

We estimated the impact of each vaccination implementation strategy over the 26 years from 2025 to 2050. Over this period, we estimated that the 2xMass18–50 strategy would have the largest epidemiologic impact, averting 490,008 additional TB cases (IQR 396,396–589,195) and 96,417 (IQR 83,779–111,250) additional TB deaths as compared to the baseline no vaccination strategy. The 2xMassAdultsHIV+ strategy would have the second largest impact, averting 367,862 (IQR 285,329–534,162) additional TB cases and 73,191 (IQR 62,485–113,424) additional TB deaths as compared to the baseline no vaccination scenario (Fig. 1 and Table 2, Supplementary Fig. 1).

**Table 2:** Cases and deaths averted, incremental costs, DALYs averted and net health benefit compared to baseline no vaccination scenario—default case.

Strategy	Cases averted	Deaths averted	Incremental cost, USD millions	DALYs averted, thousands	Mean net health benefit, DALYs averted,thousands (probability of cost-effectiveness)	
					Lower HCOC	Upper HCOC
Mass&Routine18–50	315,256	61,718	321	954	841 (0%)	874 (0%)
2xMass18–50	490,008	96,417	417	1345	1201 (65%)	1244 (70%)
Mass&RoutineAdultHIV+	209,524	42,143	41	628	712 (0%)	716 (0%)
2xMassAdultHIV+	367,862	73,191	49	948	1112 (35%)	1116 (30%)

Estimates are medians unless specified otherwise. Mass&Routine18–50—mass campaign for people aged 18–50 & routine vaccination for 18-year olds; 2xMass18–50—two mass campaigns for people aged 18–50; Mass&RoutineAdultsHIV+ mass campaign for all adults living with HIV & routine vaccination of 18-year olds living with HIV; 2xMassAdultsHIV+ two mass campaigns for all adults living with HIV. Net health benefit threshold based on health-care opportunity cost (HCOC) threshold for South Africa. Lower HCOC threshold for SA, $2,480. Upper HCOC threshold for SA, $3,334. Default-case: vaccine disease efficacy = 50%; duration of protection = 5 years; routine coverage = 40% (70% for HIV+ only campaigns); mass coverage 60%.

### Economic outcomes

Without vaccination, from 2025 to 2050, the total cumulative discounted TB-related cost (diagnosis and treatment) was estimated to be USD 1.5 billion and ART treatment was USD 17 billion, with a cumulative total cost of USD 18 billion (Supplementary Tables 2, 3 and Supplementary Fig. 1).

The 2xMass18–50 vaccine implementation strategy was the most expensive, delivering 38 million vaccine courses between 2025 and 2050, at a cumulative vaccine procurement and delivery cost of USD 507 million. The total TB-related cost under the 2xMass18–50 strategy was reduced by USD 184 million compared to the no vaccination strategy. The reduction in TB-related cost was driven by the reduction in costs for TB treatment and diagnosis, which was linked to the number of people diagnosed with TB. TB treatment cost reduced because of the lower incidence of TB under the vaccination scenario compared to no vaccination. Similarly, diagnosis cost reduced because the number of people with presumptive TB tested, which is a function of the number of people diagnosed (test-to-diagnosis ratio) and prevalence of active TB, was lower under the vaccination scenario compared to no vaccination.

The total ART cost increased by USD 79 million reflecting the fact that the reduced mortality among PLHIV compared to the no vaccination strategy increased the utilisation of ART. This is because vaccinated PLHIV not on ART had a reduced risk of TB disease and TB mortality. Therefore, the number of surviving PLHIV initiated onto ART increased, driving ART cost. Similarly, for PLHIV on ART, the reduced mortality risk increased life expectancy and thereby person-time on ART, resulting in higher ART cost compared to the no vaccination strategy. The total incremental cost of the 2xMass18–50 strategy compared to no vaccination was USD 417 million (IQR 400–433 million) (Table 2).

The Mass&Routine18–50 implementation strategy, where only one mass campaign was implemented followed by the routine vaccination of 18-year olds, delivered 29 million vaccine courses at a cumulative vaccine procurement and delivery cost of USD 374 million. The TB-related cost was reduced by USD 130 million, and the ART cost increased by 63 million, compared to the no vaccination strategy. The total incremental cost of the Mass&Routine18–50 strategy compared to no vaccination was USD 321 million (IQR 308–332 million).

The Mass&RoutineAdultHIV+ strategy, where one mass campaign was implemented followed by the routine annual vaccination of 18-year-old PLHIV, had the lowest discounted cumulative vaccine-related cost at USD 48 million, to deliver 3 million vaccine courses between 2025 and 2050. Under this strategy, TB-related cost was reduced by USD 83 million and ART cost increased by USD 72 million compared to a no vaccination strategy, and the total incremental cost was USD 41 million (IQR 33–48 million) relative to no vaccination (Table 2, Supplementary Tables 2, 3).

The 2xMassAdultHIV+ strategy, which delivered 6 million vaccine courses with USD 78 million in cumulative vaccine-related costs, resulted in the second highest TB-related cost savings of USD 133 million and the highest increase in ART cost of USD 92 million compared to no vaccination.

### Cost-effectiveness of vaccination strategies

**Fig. 2:**
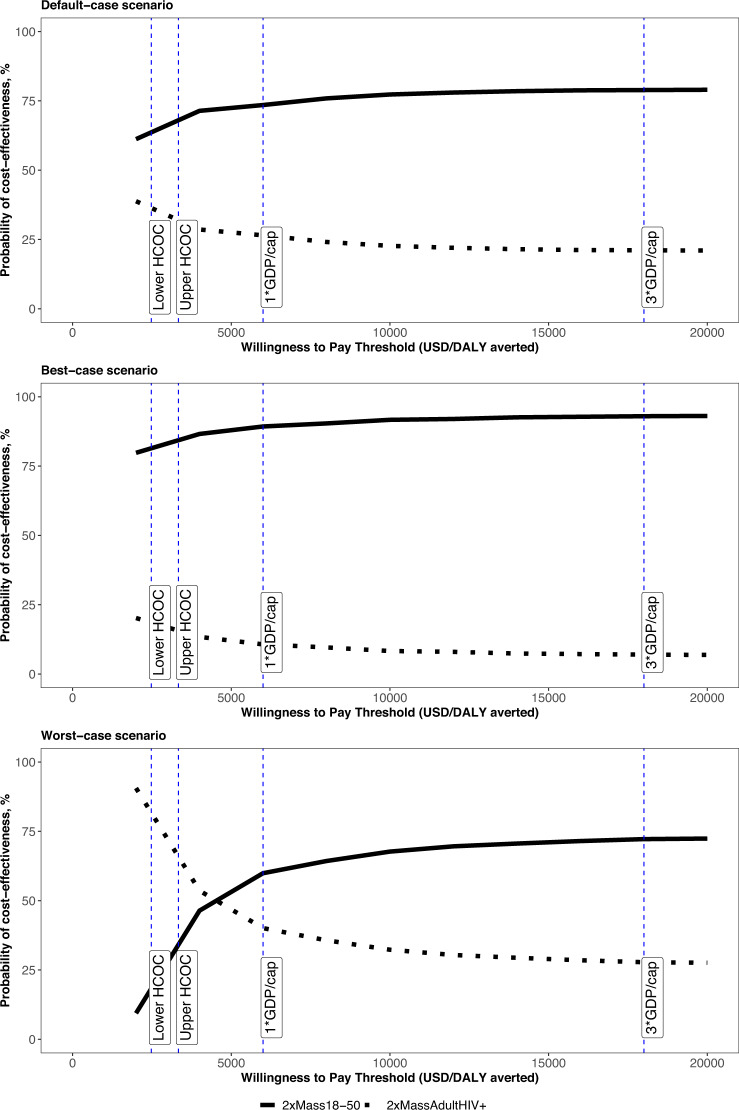
Cost-effectiveness acceptability curves for vaccination strategies 2xMass18–50 and 2xMassAdultHIV+. 2xMass18–50—two mass campaigns for people aged 18–50; 2xMassAdultHIV+—two mass campaigns for all adults living with HIV.

The 2xMass18–50 strategy was the most cost-effective implementation strategy under our default modelling assumptions, producing the largest NHB of 1.2 million DALYs averted closely followed by the 2xMassAdultHIV+ strategy with an estimated NHB of 1.1 million DALYs averted. Probabilistic sensitivity analysis characterising the parameter uncertainty in the modelling when comparing the strategies estimated the probability of the 2xMass18–50 strategy being optimal was 65% and 70% at the lower and upper HCOC thresholds respectively (Table 2 and Fig. 2). The two strategies which included an annual routine vaccination component had a 0% probability of being the most cost-effective option at these HCOC thresholds in South Africa.

The 2xMass18–50 strategy remained the most cost-effective under the best-case scenario, producing the highest NHB at the lower and upper HCOC thresholds (Supplementary Table 4), and it had the highest probability of being cost-effective in the probabilistic sensitivity analysis (Fig. 2 and Supplementary Table 4). However, under the worst-case scenario assumptions, 2xMassAdultHIV+ was the most cost-effective vaccination implementation strategy at both the lower and upper HCOC thresholds (Fig. 2 and Supplementary Table 4).

## Discussion

Our study identified a range of plausible M72/AS01_E_ vaccination implementation scenarios for South Africa and explored their feasibility by estimating their health outcomes, budget impact and cost-effectiveness. Following two rounds of modelling informed by interviews with experts, the most cost-effective expert-proposed strategy was identified as delivering two mass vaccination campaigns targeting the whole population aged 18–50, producing a NHB of 1.2 million DALYs averted with a 65–70% probability of being cost-effective. This strategy would cost USD 507 million to deliver 38 million vaccine courses, reducing TB-related cost by USD 184 million but increasing ART cost by USD 79 million. Implementing this strategy would avert 8% of the total estimated TB burden in South Africa as compared to no vaccination, suggesting that novel vaccination whilst not a silver bullet, could nevertheless be an important tool to help tackle the very substantial burden of morbidity and mortality arising from TB in the country.

As expected, targeting the whole population aged 18–50 averted more TB cases and deaths than targeting adult PLHIV, in both the mass and routine, and dual-mass campaigns, across the default, worst, and best-case scenarios. We found that the relative impact of targeting the whole population versus the adult PLHIV population was similar between best, worst, and default scenarios: whole population aged 18–50 targeted mass and routine vaccination averted approximately 50% more TB cases and deaths than adult PLHIV-targeted mass and routine vaccination, and whole population dual-mass vaccination averted approximately 30% more TB cases and deaths than PLHIV-targeted dual-mass vaccination. However, absolute differences in averted TB burden declined from best through to worst scenarios, corresponding to reduced burden averted by vaccination in each scenario. Since costs to the health system are driven by absolute TB burden, in a worse-case scenario where the vaccine was 30% effective with a 3-year duration of protection, we found the PLHIV-targeted dual-mass vaccination campaign to be the most cost-effective implementation scenario for South Africa. Those expert-proposed strategies that included a routine vaccination component did not come out to be the most cost-effective strategies under any of the scenarios we assessed. This highlights the importance of considering implementation specifics when rolling out vaccination programmes as seemingly innocuous choices can have a major impact on both epidemiological and economic outcomes.

Compared to previous work, our study has three key strengths which enabled us to more precisely characterise the epidemiological and economic impact of the M72/AS01_E_ vaccine in South Africa: (1) the use of strategies elicited from expert interview; (2) modelling repeat mass vaccination campaigns and (3) targeting PLHIV for vaccination. In contrast to previous work, which has wholly assumed vaccine strategies or extrapolated from vaccine programmes for other disease, we modelled vaccination strategies elicited from expert interviews. These strategies are more likely to reflect the constraints and practicalities of vaccination implementation in South Africa. This enabled us to assess the cost effectiveness and budget impact of TB vaccination implementation strategies previously unexplored—this is the only study to explore the impact of age- and HIV-targeted vaccination in South Africa with an M72-like vaccine. Additionally, prior dynamic-model based cost-effectiveness analyses have not investigated the impact of multiple mass campaigns^16^, or combined mass campaigns with routine vaccination. In this study, we characterised the relative impact of these strategies, finding repeat mass vaccination to be more cost-effective than routine vaccination.

We calibrated TB epidemiology to best available historical data. In addition, we included a stratum of TB in PLHIV, calibrated to disease burden in this population group, with TB natural history and outcomes parameterised to reflect the impact of HIV. This enabled us to specifically explore the impact of targeting HIV-positive populations, using targeting scenarios suggested by in-country experts. The HIV-positive stratum included ART use, allowing us to estimate changes in ART use caused by vaccine mediated reductions in TB-HIV mortality.

The limitations in this model stem principally from gaps in data. Firstly, there are no empirical data to parameterise the efficacy of an M72/AS01_E_-like vaccine in PLHIV, at any stage of HIV, whether receiving ART or not. To mitigate this gap, we varied vaccine efficacy in the sensitivity analyses and found that our findings remained robust to variation. Secondly, we assumed instant scale up of mass vaccination campaigns. In reality, scale up is likely to occur over a number of years with preliminary groundwork and preparation required to achieve the assumed coverage levels; this assumption likely overestimated vaccine impact and cost in the short-medium term. However, by extending the model time horizon to 26 years, this limitation is somewhat mitigated. Finally, we did not find examples of previous large mass vaccination campaigns in South Africa in the literature at the time this study was conducted, and the expert interviews confirmed prior mass vaccination campaigns had not been implemented in South Africa. Therefore, the health care resources utilised for the M72/AS01_E_ vaccination strategies had to be estimated based on previous immunisation activities carried out on a smaller scale in South Africa. These estimates do not account for regional variations in health care access or resource availability. However, we incorporated the infrastructure and logistical resource scale-up requirements for mass vaccine delivery based on estimates for the COVID-19 vaccination campaign targeting all adults. The on-going COVID-19 national vaccination programme in South Africa would provide lessons to further inform TB vaccination strategies. In particular, issues related to vaccine hesitancy^17,18^ would undoubtedly be a significant challenge to overcome in order to achieve the assumed vaccination coverage levels modelled in our study.

This work has important implications for global and country decision makers. It has shown that a suitably designed TB vaccination programme using the M72/AS01_E_ vaccine is likely to be highly cost-effective and affordable given the resource and budget constraints in South Africa. Furthermore, it has shown that engaging with key country stakeholders early on in the vaccine development process is an important step in order to identify feasible and affordable vaccination scenarios. With a number of novel TB vaccines in late-stage clinical trials, the type of work carried out in this study should be replicated over a much larger number of high TB burden countries, and for other candidates, using more sophisticated models able to support greater intervention detail, in order to best inform country level decision making regarding the potential introduction of these novel TB vaccines. Such work will contribute modelling evidence on the full value proposition of TB vaccines that can be used in advocacy with global and country funders in order to facilitate the rapid adoption of these new vaccines in countries where they are shown to be affordable and to provide good value for money.

## Methods

### Implementation strategies

In order to determine implementation strategies and target groups in South Africa, we conducted two rounds of interviews with expert decision makers/actors between May and December 2020. Participants were chosen based on their expertise (TB, vaccines, vaccine policy, vaccine supply, and vaccine delivery), sector (civil society, academic, Ministry of Health, Ministry of Finance, or membership in a non-governmental organisation), and scope (national, regional, and local level). Further details of the interview process and summary of responses are described in Pelzer et al.^19^, Supplementary Note 1 and Supplementary Table 5.

In the first round of interviews, with eight experts we explored vaccination implementation strategies that in their opinion had a high likelihood of succeeding in South Africa. We subsequently structured these proposed strategies into epidemiological scenarios that could be mathematically modelled to generate implementation strategies based on target population, coverage, vaccination schedule and delivery mode. In the second round of interviews, we presented these structured vaccination strategies to the interviewees to elicit constraints and preferences, and rule out infeasible strategies. We then refined our modelling to reflect this feedback and projected TB cases and deaths from 2025–2050, and estimated the cost-effectiveness and budget impact of the feasible vaccination implementation strategies identified.

### Modelling approach

We adapted a published age-stratified compartmental difference-equation based dynamic transmission model of *Mycobacterium tuberculosis* (*M. tb*) transmission calibrated to demographic and epidemiologic data from South Africa^13^. We stratified the tuberculosis natural history parameters of the model by age-group (children, age ≤14; and adults, age ≥15) and HIV-status, where appropriate. Individuals with HIV were assumed to have higher rates of progression to active TB disease, reactivation from latent TB, relapse after recovery from TB, TB mortality, and lower protection against progression to active disease following reinfection with *M. tb*^20–25^. Full details of the model structure, equations, parameterisation, and calibration are given in Harris et al.^13^ and the Supplementary Note 2.

We calibrated the model to epidemiologic data from South Africa between 2000 and 2016 and projected model results over 35 years from 2016 to 2050. The underlying demographic structure was calibrated to age-stratified data from World Population Prospects^26^. Epidemiologic calibration targets included overall rates (per 100,000 population) of TB incidence, notifications, bacteriologically-positive prevalence, and mortality. Incidence and notification rates were also calibrated by age for children (age ≤14 years) and adults (age ≥15 years). We constrained TB-HIV coinfection through calibration to overall rates of TB incidence, and TB notifications and mortality in HIV-positive populations.

To propagate uncertainty through the model, we sampled values for model natural history parameters using Approximate Bayesian Computation Markov Chain Monte Carlo using prior ranges derived from the literature^4,15^. We randomly subsampled 1000 fully calibrated parameter sets through which we generated model runs. Due to the asymmetric distributions in epidemiologic outcome projections, median model outcomes were used as estimates of central tendency, with the measure of dispersion given by the interquartile range.

Based on input received from the interviews with key experts and the M72/AS01_E_ clinical trial results, for the default vaccination scenario, we simulated a prevention of disease vaccine conferring 50% efficacy for 5 years, effective in individuals with a previous history of *M. tb* infection (‘post-infection vaccine’)^6^. The vaccine was assumed to be “leaky”, whereby vaccinated individuals could still progress to active disease, albeit at a rate reduced in proportion to the efficacy of the vaccine. Vaccine coverage was represented as the proportion of the target population in the vaccinated stratum within the model. Coverage was held constant during the period of vaccine efficacy. We assumed instantaneous scale of vaccine coverage to the target value, and vaccine waning assumed to occur instantly at the end of the duration of protection. The efficacy of M72/AS01_E_ in PLHIV has not yet been established. However, prior phase I/II studies have demonstrated safety and immunogenicity (M72-specific cellular and humoural immune responses) in PLHIV^27,28^. On this basis, we assumed that M72/AS01E was likely to be effective in PLHIV. As efficacy data are lacking, we varied the vaccine efficacy in PLHIV in the sensitivity analysis. We assumed a vaccine course comprised of two vaccine doses. For the default scenario, we estimated 60% mass and 40% routine vaccination coverage, respectively. Target coverage and scale up of mass vaccination were assumed to be achieved instantly at vaccine deployment.

We calculated vaccine impact by comparing the model outputs in simulations with vaccination implemented according to each of the vaccination implementation strategies identified compared to counterfactual unvaccinated simulation output. Our primary modelled outcome measures were the number of TB cases and deaths over a 26-year time-horizon (2025–2050) which we then used to estimate a range of health and economic outcomes.

### Summary health outcomes

Outcomes were estimated as the median values of the transmission model runs with the 1000 randomly subsampled parameter sets. We used the modelled estimates for deaths due to TB by age and year, and time spent with active TB by HIV status and combined these with disability weights for active TB by HIV status^29^ and life expectancy by age-group from the UN Population Division^26^ in order to calculate health outcomes associated with each strategy in terms of Disability Adjusted Life Years (DALYs) averted^30^. DALYs averted were then calculated for each of the vaccination strategies by comparing health outcomes with the counterfactual no vaccination strategy. Future DALYs averted were discounted at 3%, as recommended in the International Decision Support Initiative (iDSI) reference case^31^.

### Cost estimation and budget impact

We estimated the corresponding costs for the outcomes generated from the transmission model runs with the 1000 randomly subsampled parameter sets and reported the median values. We estimated costs from the South African health system perspective in 2019 values, the latest year for which the World Bank GDP deflator for South Africa was available at the time of analysis, and converted to US dollars (USD) based on official International Monetary Fund exchange rates^32^. We searched electronic databases (Medline, EBSCO, Cochrane library, CINAHL, EconLit) and conducted interviews with local experts to identify key resources required to deliver the vaccination implementation strategies and collate unit costs for these resources. We divided cost categories into vaccine procurement, vaccine delivery, TB-related and ART treatment costs (Table 3). Quantities of resources used were based on the epidemiological model estimates for the number of TB treatment initiations, number of TB tests conducted, and number of two-dose vaccine courses delivered (Supplementary Table 6). The number of TB tests conducted was calculated using an estimated Test-to-Diagnosis (TDR) ratio of 12.8^33^ and this value was adjusted annually by the prevalence of active tuberculosis in South Africa. We assumed 5 min of vaccinator time per dose and 45 work hours per week to estimate the number of vaccinators required. We estimated incremental costs as the difference in total cost between each of the vaccine implementation strategies and the baseline no vaccination strategy. We used a 3% discount rate, in line with the iDSI reference case, to convert future costs to 2019 values^31^.

**Table 3:** Summary of economic parameters used.

Economic parameters	Value^a^	Source	Notes
TB-related unit costs			
DSTB diagnosis, per patient	26.52	32	Cost per Xpert test
DSTB treatment, per episode	185.86	32	Category 1 treatment episode
Vaccine procurement			
Vaccine price, per course	10	Expert opinion	Two doses per course. 5% wastage assumed.
Freight, per dose	1	Assumed	10% of vaccine price
Vaccine delivery—mass campaign			
Social mobilisation, per dose	0.08	33	
Cold chain (service level), per dose	0.01	34	
Transport(facility-based), per dose	0.04	34	
Transport for outreach services, per dose	0.004	33	
Vaccinator cost, per dose	0.69	35,36	5 minutes of vaccinator time per dose assumed. Vaccinator cost based on the 2019 median annual salary of a professional nurse in South Africa.
Per diem for outreach service delivery, per dose	1.39	34	Per diems and allowances paid to vaccinators for service delivery during outreach activities. Estimated median value based on previous vaccination campaigns
Vaccine delivery—routine			
Cold chain (service level), per dose	0.01	34	
Transport(facility-based), per dose	0.04	34	
Social mobilisation, per dose	0.08	33	
Vaccinator cost, per dose	0.69	35,36	5 minutes of vaccinator time per dose assumed. Vaccinator cost based on the 2019 median annual salary of a professional nurse in South Africa.
ART cost per patient year	254	37	
Upper health care opportunity cost threshold for South Africa (USD)	2480	38	
Lower health care opportunity cost threshold for South Africa (USD)	3334	38	
1-times GDP per capita South Africa 2019	6001	39	
3-times GDP per capita South Africa 2019	18,004	39	

^a^Unit costs in 2019 USD. 31% health system mark-up applied to vaccine delivery cost.

We calculated feasibility and budget impact estimates for each vaccine implementation strategy by estimating the total quantities of resources required to deliver each vaccine implementation strategy across the entire target population and applied unit costs to these estimates in order to calculate budget impact in monetary terms.

### Cost-effectiveness

For the vaccination strategies, the DALYs averted were combined with the costs of the vaccination programme, ART cost and any reduction in TB-related costs from vaccination to calculate net health benefits (NHB) using the upper and lower health care opportunity cost (HCOC) thresholds for South Africa estimated by Ochalek et al. which ranged between USD 2,480 and USD 3334 per DALY averted^34^. NHB was calculated in terms of DALYs averted as: net health benefit = DALYs averted − (incremental cost/health-care opportunity cost threshold).

### Characterising uncertainty

Alongside our default scenario of 50% vaccine efficacy, 5 years duration of protection and 60% mass campaign coverage, we also conducted scenario analyses for best and worst-case scenarios pertaining to vaccine characteristics and vaccination coverage. Under a best-case scenario, we modelled the vaccine with a 70% efficacy (the upper bound of the 90% confidence interval for vaccine efficacy in the M72/AS01_E_ clinical trial)^6^ and assumed a 10-year duration of protection and 80% mass campaign coverage. For the worst-case scenario, we modelled a 3-year duration of protection (the length of the M72/AS01_E_ PhIIb clinical trial follow-up period)^6^ and assumed 30% vaccine efficacy with only 40% mass campaign coverage. Under all three scenarios, we assumed routine campaign coverage of 40%, based on expert feedback from the interviews.

We ran probabilistic sensitivity analysis on our results to capture uncertainty in our parameter estimates of mortality, prevalence, bacteriologically-positive prevalence, TB notifications, number of people vaccinated and number of people on ART. For each of the 1000 model runs, we estimated the cases and deaths averted, DALYs averted, incremental costs, and the NHB values for the vaccination strategies relative to no vaccination. We subsequently calculated the probability of each vaccination strategy producing the highest NHB for each of the health care opportunity cost thresholds considered across the 1000 model runs.

### Reporting summary

Further information on research design is available in the Nature Research Reporting Summary linked to this article.

## Supplementary information

Supplementary Information

REPORTING SUMMARY

## Data Availability

Detailed model output data and model code available on request.
