# Supplementary material for: Feasibility of novel adult tuberculosis vaccination in South Africa: a cost-effectiveness and budget impact analysis
Source: NPJ Vaccines. 2022 Nov 7;7:138. doi: 10.1038/s41541-022-00554-1 (PMC9640704; doi:10.1038/s41541-022-00554-1)
Supplement: Supplementary file 1 — Supplementary Information [file 41541_2022_554_MOESM1_ESM.pdf]

Feasibility of novel adult tuberculosis vaccination in South Africa: a cost-effectiveness and budget impact analysis

Sahan Jayawardana, Chathika K. Weerasuriya, Puck T. Pelzer, Janet Seeley, Rebecca C. Harris,

Michele Tameris, Dereck Tait, Richard G White, Miqdad Asaria

Supplementary Information

Supplementary Table 1: Expert-proposed vaccination strategies identified from interviews

Vaccination strategies identified from the first round of interviews	Constraints and preferences identified from the second round of interviews
Annual routine vaccination targeting adolescents aged 12-14 years implemented through primary and high schools.	Epidemiological impact of vaccine could be limited due to the young age of the target group. M72/AS01$_E$ clinical trial only enrolled adults 18-50 years of age – no clinical evidence for younger age groups.
Mass vaccination campaign targeting adolescents below the age of 16 implemented in schools.	Epidemiological impact of vaccine could be limited due to the young age of the target group. M72/AS01$_E$ clinical trial only enrolled adults 18-50 years of age – no clinical evidence for younger age groups.
Mass campaign targeting people aged 18-35 years followed by the routine vaccination of 18-year-olds implemented via colleges, universities and workplaces.	Implementing vaccination only through workplaces will exclude many people in this age group – for example, unemployed people. A vaccination strategy implemented through workplaces and further outreach services, targeting people aged 18-50, was preferred.
Mass campaign targeting people aged 16-35 followed by the routine vaccination of 16-year-olds delivered through local clinics.	M72/AS01$_E$ clinical trial only enrolled adults 18-50 years of age – no clinical evidence for younger age groups. A vaccination strategy implemented through workplaces, local clinics and further outreach services, targeting people aged 18-50, was preferred.
Mass campaign targeting all adults.	A campaign targeting the entire adult population of South Africa was not viewed as feasible due to capacity constraints, cost and low adult coverage achieved during previous targeted vaccination campaigns (e.g., influenza)
Vaccination campaign targeting all PLHIV delivered from clinics providing antiretroviral therapy	A campaign targeting PLHIV was viewed as feasible due to the targeted clinic-based approach and the high TB burden amongst this population. However, there could be sensitivities and privacy concerns around targeting PLHIV due to social stigma.

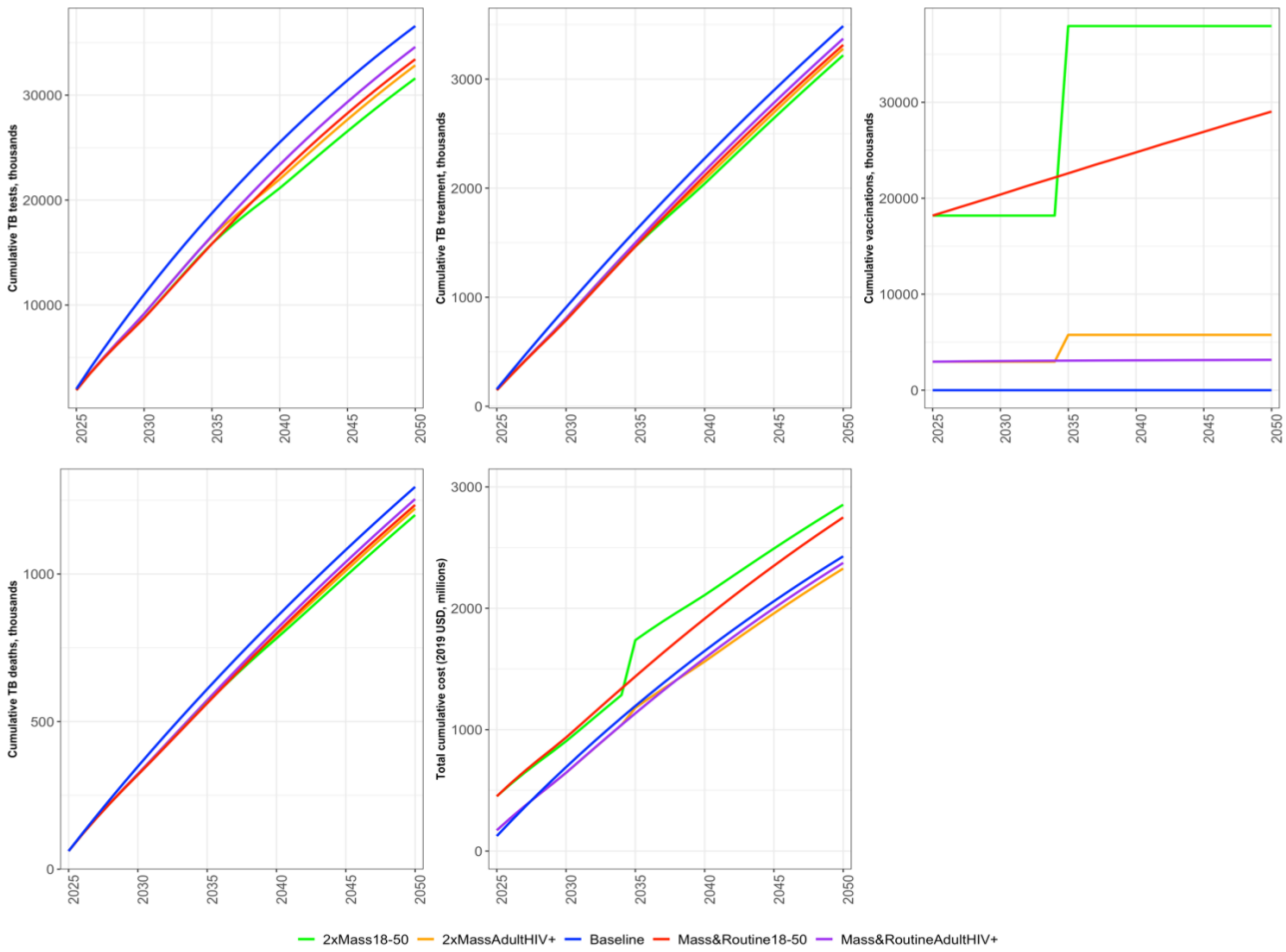

Supplementary Figure 1. Cumulative outcomes and resource use estimates from 2025 to 2050 for each vaccine implementation scenario. Total cost comprises of TB-related and vaccine related costs.

Mass&Routine18-50 – mass campaign for people aged 18-50 & routine vaccination for 18-year-olds; **2xMass18-50** – two mass campaigns for people aged 18-50; **Mass&RoutineAdultsHIV+** - mass campaign for all adults living with HIV & routine vaccination of 18-year-olds living with HIV; **2xMassAdultsHIV+** - two mass campaigns for all adults living with HIV.

Supplementary Table 2: Cumulative costs for M72/AS01$_E$ vaccination implementation in South Africa: 2025 – 2050 - health system perspective (2019 USD), default case

		Baseline (no vaccine)	Mass&Routine18-50	2xMass18-50	Mass&RoutineAdultHIV+	2xMassAdultHIV+
TB-related cost	DS-TB diagnosis	1,466,383,717	1,332,441,948	1,260,169,412	1,392,223,386	1,328,814,852
	DS-TB treatment	982,485,443	936,897,611	909,197,056	949,640,405	926,087,146
Vaccine-related cost	Procurement cost	NA	365,880,950	478,259,253	39,843,324	72,665,069
	Delivery cost	NA	128,748,712	220,151,085	17,632,495	33,449,000
ART costs		28,017,566,248	28,146,166,831	28,176,697,784	28,154,819,473	28,220,035,898
Total		30,412,047,172	30,865,033,587	31,005,287,699	30,505,590,111	30,514,096,411

Mass&Routine18-50 – mass campaign for people aged 18-50 & routine vaccination for 18-year-olds; **2xMass18-50** – two mass campaigns for people aged 18-50; **Mass&RoutineAdultsHIV+** - mass campaign for all adults living with HIV & routine vaccination of 18-year-olds living with HIV; **2xMassAdultsHIV+** - two mass campaigns for all adults living with HIV.

Supplementary Table 3: Total discounted costs for M72/AS01$_E$ vaccine implementation in South Africa: 2025 – 2050; health system perspective (2019 USD)

	Baseline (no vaccine)	Mass&Routine18-50	2xMass18-50	Mass&RoutineAdultHIV+	2xMassAdultHIV+
TB-related cost	1,505,029,222	1,374,691,196	1,320,586,469	1,422,166,870	1,371,605,107
Vaccine-related cost	NA	373,966,823	506,979,423	47,559,751	77,882,373
ART costs	16,871,014,218	16,934,293,462	16,949,762,177	16,942,949,819	16,962,877,530
Total cost	18,357,369,793	18,679,330,754	18,773,450,292	18,399,482,277	18,403,648,984

Mass&Routine18-50 – mass campaign for people aged 18-50 & routine vaccination for 18-year-olds; **2xMass18-50** – two mass campaigns for people aged 18-50; **Mass&RoutineAdultsHIV+** - mass campaign for all adults living with HIV & routine vaccination of 18-year-olds living with HIV; **2xMassAdultsHIV+** - two mass campaigns for all adults living with HIV.

Supplementary Table 4: Cases and deaths averted, incremental costs, DALYs averted and mean net health benefit compared to baseline no vaccination scenario – default, best and worst-case scenarios

Strategy	Cases averted	Deaths averted	Incremental cost, USD millions	DALYs averted	Mean net health benefit, DALYs averted (probability of cost-effectiveness)	
					Lower HCOC	Upper HCOC
**Default case**						
**Mass&Routine18-50**	315,256	61,718	321	954,914	840,543 (0%)	873,755 (0%)
**2xMass18-50**	490,008	96,417	417	1,345,447	1,201,253 (65%)	1,244,412 (70%)
**Mass&RoutineAdultHIV+**	209,524	42,143	41	628,027	711,698 (0%)	715,964 (0%)
**2xMassAdultHIV+**	367,862	73,191	49	948,166	1,111,626 (35%)	1,116,325 (30%)
**Best-case scenario**						
**Mass&Routine18-50**	1,050,476	205,932	297	2,966,151	2,915,832 (0%)	2,946,685 (0%)
**2xMass18-50**	1,638,701	323,834	396	4,205,511	4,158,908 (83%)	4,199,715 (85%)
**Mass&RoutineAdultHIV+**	682,439	136,355	24	1,886,192	2,217,033 (0%)	2,219,405 (0%)
**2xMassAdultHIV+**	1,210,150	237,299	-10	2,864,027	3,496,674 (17%)	3,494,522 (15%)
**Worst-case scenario**						
**Mass&Routine18-50**	81,345	16,109	267	255,612	150,022 (0%)	177,668 (0%)
**2xMass18-50**	122,527	24,085	316	347,558	225,793 (17%)	258,479 (35%)

**Mass&RoutineAdultHIV+**	53,967	10,884	32	167,228	179,546 (0%)	182,921(0%)
**2xMassAdultHIV+**	94,109	18,791	46	251,392	277,831 (83%)	282,524(65%)

Notes: Estimates are medians unless specified otherwise.

Mass&Routine18-50 – mass campaign for people aged 18-50 & routine vaccination for 18-year-olds; **2xMass18-50** – two mass campaigns for people aged 18-50; **Mass&RoutineAdultsHIV+** - mass campaign for all adults living with HIV & routine vaccination of 18-year-olds living with HIV; **2xMassAdultsHIV+** - two mass campaigns for all adults living with HIV.

Net health benefit threshold based on healthcare opportunity cost (HCOC) threshold for South Africa. Lower HCOC threshold for SA, $2,480. Upper HCOC threshold for SA, $3,334.

Default-case: vaccine disease efficacy = 50%; duration of protection =5 years; routine coverage = 40% (70% for HIV+ only campaigns); mass coverage 60%.

Best-case scenario: vaccine disease efficacy = 70%; duration of protection =10 years; routine coverage = 40% (70% for HIV+ only campaigns); mass coverage 80%.

Worst-case scenario: vaccine disease efficacy = 30%; duration of protection =3 years; routine coverage = 40% (70% for HIV+ only campaigns); mass coverage 40%.

Supplementary Note 1

Interview Guide

Section 1: Semi-structured questions

The purpose of this section is to explore/understand the acceptability of potential new TB vaccines, some of the potential challenges/barriers to introducing new TB vaccines for adolescents and/or adults, and to identify possible solutions. For interviewees in programme financing there is also discussion around prioritisation of financing.

Acceptability

1. If an efficacious vaccine were available, do you think there would be interest in a new TB vaccine at the national/regional/individual level?
    1. And would there be any differences between the M72/AS01E and BCG revaccination?

2. If there were 3-4 pieces of data you consider critical to your country/program's ability to make decisions on TB vaccine introduction, what would those be? (*E.g., Efficacy in specific high-risk group, clinical studies in local context; cost of delivery to adolescents, impact on TB burden if given to different target groups etc.)*

Challenges/barriers/solutions

3. Are there any aspects with regards to **possible characteristics of the vaccine** that you think are particularly important or could be problematic? Are there any aspects that could be a deal breaker?
    1. What do you think could be done to help overcome these?

4. Are there any potential **political** barriers to introducing an adult/adolescent TB vaccine?
    1. What do you think could be done to help overcome these?

5. Are there any potential **financial** barriers to introducing an adult/adolescent TB vaccine?
    1. What do you think could be done to help overcome these?

6. Are there any potential **logistical** barriers to introducing an adult/adolescent TB vaccine?
    1. What do you think could be done to help overcome these?

7. Are there any potential **acceptability barriers** at the individual or community level to introducing an adult/adolescent TB vaccine?
    1. What do you think could be done to help overcome these?

8. What challenges might be faced in introducing **routine** vaccination?
    1. What do you think could be done to help overcome these?

9. What challenges might be faced in introducing **mass** vaccination?

    a. What do you think could be done to help overcome these?

10. Are there any challenges or barriers associated with **adding a vaccine to the existing immunisation programme**? (e.g. capacity, resources)
    1. What do you think could be done to help overcome these?

11. Are there any **other** challenges or barriers to introducing an adult/adolescent vaccine that you think might be important?
    1. What do you think could be done to help overcome these?

12. If those changes could be put into place, what impact do you think they would have on the introduction of the vaccine (e.g. more likely to be introduced, better coverage, better acceptance at the national/community/individual level)?

Section 2: Research questions

We will continue with structured questions on the possible delivery of an adult/adolescent TB vaccines. Modelling of new TB vaccines suggests that greater and faster impact would be achieved by vaccinating adolescents/adults, instead of children, with new TB vaccines. Therefore, we are trying to understand how new TB vaccines might be delivered to adolescents/adults and the associated costs of delivery. We will hereby focus on the M72/AS01E candidate and BCG revaccination

Did you have any questions about the background information we send about the M72/AS01E candidate and BCG revaccination

13. For the M72/AS01E /AS01E vaccine:

    a) Which populations would you vaccinate (e.g. age groups, risk groups, everyone ?)

    b) What do you anticipate the rough population size is? (1000s, 10 000s, 100 000s)

    c) Would you use routine vaccination (at a given time or age), or mass campaigns to vaccinate this group

    d) How and where would the vaccine be delivered as described above? (e.g. at existing interaction with the health service, school, work place, community campaigns)

    e) Which of these groups would be prioritized in roll-out?

For the target groups identified in question 1a-e: focus on all-pop, age-related, HIV status and SES, focus biggest population, high priority risk group:

f) How much time after registration / policy recommendation expect to start

g) What coverage do you think would be achieved (for routine maintained coverage, for mass peak coverage)?

h) How quickly would that coverage be achieved?

i) For mass, what would be the frequency and length of campaigns (years)

j) How much do you think it would be reasonable to spend per year on a TB vaccination program like this? How have you come to this amount?

k) What is the % of GDP

l) From which budget does this come

14. Regarding M72/AS01E : If the vaccine is effective for 10 years, how would that change any of your answers?

15. For revaccination with the BCG vaccine:

m) Which populations would you vaccinate (e.g. age groups, risk groups, everyone ?)

n) What do you anticipate the rough population size is? (1000s, 10 000s, 100 000s)

o) Would you use routine vaccination (at a given time or age), or mass campaigns to vaccinate this group

p) How and where would the vaccine be delivered as described above? (e.g. at existing interaction with the health service, school, work place, community campaigns)

q) Which of these groups would be prioritized in roll-out?

For the target groups identified in question 1a-e: focus on all-pop, age-related, HIV status and SES, focus biggest population, high priority risk group:

r) How much time after registration / policy recommendation expect to start

s) What coverage do you think would be achieved (for routine maintained coverage, for mass peak coverage)?

t) How quickly would that coverage be achieved?

u) For mass, what would be the frequency and length of campaigns (years)

v) How much do you think it would be reasonable to spend per year on a TB vaccination program like this? How have you come to this amount?

w) What is the % of GDP

x) From which budget does this come

16. Regarding BCG revaccination: If this will be found effective for Mtb infected (IGRA+) individuals, how would that change your answers?

For the following questions, if the question is outside of the area of expertise/knowledge of the interviewee, note this down and skip to the next question.

Tuberculosis/HIV control programmes (only for participants answering yes to tuberculosis experience)

17. Do you think there will be changes in the TB control programme in the future (e.g. changes to treatment regimens or other control measures)? If so, what do you think will change, how do you think it will change and when?

18. Do you think there will be changes in the HIV control programme in the future (e.g. changes to treatment regimens or other control measures)? If so, what do you think will change, how do you think it will change and when?

19. How do you think coordination between TB and vaccine programs will work on policy, funding and execution?

Experience with existing adult/adolescent vaccines

20. Has a new vaccine for adolescents and/or adults been introduced in to the vaccination schedule in South Africa)? If yes, which vaccine, who was it given to, how was it delivered to that population, and what coverage was achieved? If no, skip to Section 2-.

21. Can you describe some of the logistical details of how the vaccine was introduced and delivered? If no, skip to Q8. If yes, can you describe the costs of the resources and services you just described? If you do not know the cost, do you know where this data could be found?

22. Which budgets did these come from (i.e. who was the payer for the different items)?

23. How much was/is spent per year on this vaccination program?

24. If the interviewee described the introduction of a vaccine for adolescents/adults in the previous questions: what were some of the challenges/barriers to introducing that vaccine? Would those same challenges be faced with the introduction of an adult/adolescent TB vaccine?

Prioritisation of financing

25. Are you able to share the approximate governmental budget for the TB programme, the immunisation programme, and/or the HIV programme? If you do not know the budget, do you know where this data could be found?
26. % GDP

27. Who would need to give approval for reallocation of existing funding or allocation of new funding to cover the resource requirements of this program? What do you think they would consider in making this decision?

28. When thinking about a potential budget for a TB vaccine you consider impacts on other areas beyond the direct costs and benefits of this program when thinking about how much to spend? *(For example:*

- *Reduced treatment costs of recipients in the future*
- *Impacts on other disease areas e.g. if HIV patient is prevented from dying from TB then have to treat patient for HIV for longer*
- *Impacts on other members of the family of the treated patient e.g. financial impoverishment, caring time and effort etc.*
- *Impacts on the wider economy in terms of the economic contribution of a healthy population*
- *Any other impacts)*

29. If economic impact estimations are available who would we best run those by?

Regulatory process

30. Would it be possible to seek a fast-track regulatory process for approval of the vaccine, once available? And if so would you seek this type of process?

Wrap-up

31. Is there anything you would like to add that you did not have a chance to say?

Supplementary Table 5: Summary of issues identified from semi-structures interviews regarding the introduction of the M72/AS01$_E$ vaccine in South Africa

Theme	Summary of responses from interviewees
Acceptability	▪ Strong political will to implement new adult TB vaccination due to high TB and HIV burden<br>▪ No strong anti-vaccination movement in South Africa<br>▪ Civil society groups in place to help increase vaccine uptake<br>▪ Ability to piggyback on existing programme(s) would be preferred by decision-makers
Key information to help decision-makers	▪ Evidence on safety and efficacy in high-risk groups such as PLHIV<br>▪ Cost-effectiveness of the vaccination programme<br>▪ Epidemiology of TB infection and disease at the sub-national level<br>▪ Factors influencing vaccine hesitancy and coverage rates
Barriers to introduction of vaccine	▪ Reluctance to take-up vaccine due to lack of knowledge and education of TB<br>▪ Even though vaccine hesitancy historically not an issue in South Africa, misinformation around vaccination for COVID-19 may have generated elements of vaccine hesitancy<br>▪ Financial constraints – cost of the vaccine, cost of the adjuvant, cost of mass vaccination campaigns<br>▪ Manufacturing and availability of vaccine if not profitable to manufacturers<br>▪ Logistical issues arising from a 2-dose vaccine – lack of capacity to follow-up people for second dose, limitations with existing information technology infrastructure<br>▪ Any testing requirements(TB or HIV) for vaccination would add further constraints due to logistical and cost barriers<br>▪ Capacity for outreach and mobile vaccination limited - reliance on individuals coming to the health system
Steps to help overcome barriers	▪ Engage civil society to increase coverage<br>▪ Understand and address vaccine hesitancy<br>▪ Local vaccine manufacturing could increase ownership and uptake<br>▪ Set up information technology infrastructure for immunisation monitoring<br>▪ Single dose vaccine would simplify administration
Target population	▪ Adolescents, PLHIV, high risk groups(living in high density area, mining sector , unemployed, migrants), health care workers, adults in general population
Vaccination delivery options	▪ School based immunisation programme<br>▪ Vaccination at health clinics<br>▪ Vaccination at colleges, universities and workplaces<br>▪ Vaccination via clinics providing antiretroviral therapy<br>▪ Vaccination upon health visits
Expected coverage	▪ Possible to achieve above 70% coverage if vaccination integrated into existing programme(e.g., HPV vaccination programme or ART)<br>▪ Coverage will be lower for mass vaccination campaigns targeting general population (40% - 60%)

Notes: TB=tuberculosis; HIV=human immunodeficiency virus, PLHIV=people living with HIV, HPV= human papillomavirus, ART= antiretroviral therapy

Supplementary Note 2

Summary Model Description

An age-stratified population-level compartmental deterministic transmission model, calibrated to the TB epidemics in China, India and South Africa was developed in R[1] . A full description of the model structure, equations, parameterisation, and calibration are given in Harris et al[2].

The model includes five TB natural history states: susceptible, latent, bacteriologically-positive active disease, bacteriologically-negative active disease, and recovered from disease. Each compartment was represented by an unvaccinated and a vaccinated stratum. Age was modelled in single years from 0–100 years. Each natural history state was also modelled with an HIV-positive and HIV-negative stratum.

Age-specific background mortality was applied to all infection states. AIDS mortality (uH) was only applied to HIV-positive populations. A proportion of individuals experienced fast progression directly to primary TB disease following infection. The remainder of those infected entered latency. Latently infected individuals could develop active disease through 'slow progression' of the existing infection or 'fast progression' upon reinfection . Some protection against development of disease was assumed to be provided by the immune response to existing or past infection. For all new active cases, regardless of whether fast or slow progressors, a proportion developed bacteriologically positive active disease, and the remainder developed bacteriologically-negative disease. Detection (parameterised as the case detection ratio, CDR) and effective treatment (CoT) of active cases moved new cases directly to the recovered state. Undetected cases entered the relevant active disease state and became prevalent cases. Prevalent cases could be removed by natural cure (n) to the recovered state, TB death, all-cause mortality, and by AIDS-related mortality in HIV-positive populations. Individuals with bacteriologically negative disease could convert to infectious disease. The population in the recovered state could

be re-infected to develop primary active disease or enter the latent pool. Reactivation/relapse to one of the active disease states was also possible from the recovered state, at a higher rate than reactivation from the latent state. HIV infection could occur in any TB natural history state, and incident HIV in 0-4 year olds was assumed to occur at birth by mother to child transmission.

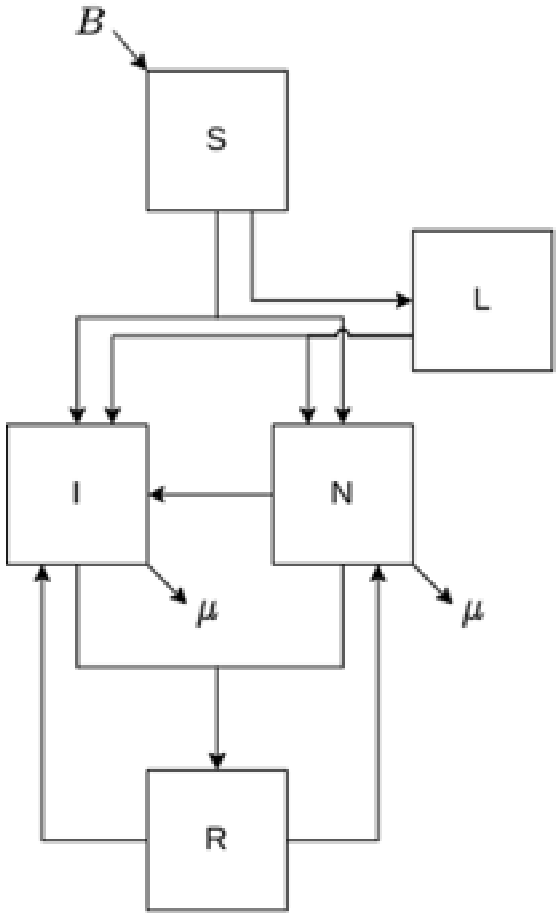

Supplementary Figure 2: Summary of Model Structure. Boxes represent compartments, arrows represent flows between compartments. Background (non-TB) mortality was applied to all compartments. Mortality due to TB was applied to the I and N compartments (μ), births entered the S compartment (B). S=susceptible; I=infectious active; N=noninfectious active; L=latent infection; R=recovered.

Supplementary Table 6: Resource use for M72 vaccine implementation in South Africa: 2025-2050, default case

	**Baseline (no vaccine)**	**Mass&Routine18-50**	**2xMass18-50**	**Mass&RoutineAdultHIV+**	**2xMassAdultHIV+**
No. of TB treatment initiations[a]	3,523,979	3,360,465	3,261,109	3,406,171	3,321,690
No. of TB tests[b]	36,843,812	33,478,441	31,662,548	34,980,487	33,387,308
No. of vaccine courses procured[c]	-	30,490,079	39,854,938	3,320,277	6,055,422

Mass&Routine18-50 – mass campaign for people aged 18-50 & routine vaccination for 18-year-olds; **2xMass18-50** – two mass campaigns for people aged 18-50; **Mass&RoutineAdultsHIV+** - mass campaign for all adults living with HIV & routine vaccination of 18-year-olds living with HIV; **2xMassAdultsHIV+** - two mass campaigns for all adults living with HIV.

[a] Number of total TB notifications from 2025 – 2050 estimated by transmission model.

[b] Number of people tested was calculated using an estimated Test-to-Diagnosis (TDR) ratio for South Africa in 2025. A TDR value of 12.8 was used based on Xpert test (primary diagnostic tool used in South Africa) results data from the South Africa National Health Laboratory Services. This value was adjusted for subsequent years by the prevalence of active tuberculosis.

[c] A course was assumed to comprise of two vaccine doses

References

1. R Core Team. A language and environment for statistical computing. (2022).
2. Harris, R. C., Sumner, T., Knight, G. M., Zhang, H. & White, R. G. Potential impact of tuberculosis vaccines in China, South Africa, and India. *Sci Transl Med* **12**, (2020).
